# Maintained diabetes remission among normal BMI individuals achieved without ongoing intervention: a three-year follow-up study of intermittent calorie restriction

**DOI:** 10.3389/fendo.2025.1733840

**Published:** 2026-01-28

**Authors:** Ruiyu Wu, Xiao Yang, Xu Zhou, Zhiyong Xiao, Xuan Chen, Jiali Zhou, Jian Li, Zhuoming Yin, Xihu Lai, Tao Wang, Quanmin Li, Dongbo Liu

**Affiliations:** 1 Horticulture College, Hunan Agricultural University, Changsha, China; 2 State Key Laboratory of Subhealth Intervention Technology, Changsha, China; 3 Hunan Provincial Engineering Research Centre of Medical Nutrition Intervention Technology for Metabolic Diseases, Changsha, China; 4 Nutrition and Food Catering, Changsha Commerce and Tourism College, Changsha, China; 5 Hunan Source Integration Biotechnology Co., Ltd., Yiyang, China; 6 Xincheng Smart Internet Hospital, Chengdu, China; 7 Dongyue Hospital, Shijiazhuang, China; 8 Department of Endocrinology, Peking University Shougang Hospital, Beijing, China

**Keywords:** Chinese medical nutrition therapy, diabetes complications, follow-up, intermittent calorie restriction, long-term diabetes remission, type 2 diabetes

## Abstract

**Objective:**

Even when diabetes remission is achieved and maintained through weight loss, relapse is likely if body weight and consequently BMI increase again. This study aims to evaluate the maintenance of remission without ongoing intervention among participants who achieved diabetes remission and normal BMI through intermittent energy restriction.

**Research design and methods:**

This study was a randomized controlled trial with parallel-design and open-label. The intervention group received a three-month Chinese Medical Nutrition Therapy (CMNT) diet intervention consisting of six 15-day cycles. Each cycle encompassed five consecutive days of energy restriction (840 kcal/day), succeeded by 10 days of ad libitum eating. During the ad libitum dietary phase and the subsequent 3-year follow-up, participants self-selected food according to the Chinese Dietary Guidelines for Diabetes (2017 edition). Eventually, their HbA1c, fasting blood glucose (FBG), antidiabetic medication use, weight, quality of life, and complications were assessed.

**Results:**

After 3 years of follow-up, 75% of participants in the CMNT group maintained diabetes remission compared to none in the control group. The maintenance of remission was found to be positively correlated with the extent of withdrawal of insulin or insulinotropic agent through the intervention. However, for people whose BMI is below 24 kg/m², regaining weight did not affect the maintenance of remission. Additionally, the CMNT group exhibited significant improvements in quality of life, along with lower rates of complications, hospitalizations, and all-cause mortality.

**Conclusion:**

For individuals with normal BMI, after following a three-month CMNT, diabetes remission is observed over three years without ongoing structured maintenance.

**Clinical Trial Registry:**

chictr.org.cn, identifier ChiCTR2000038036.

## Highlights

- The CMNT diet as a promising intervention for achieving long-term diabetes remission, can maintain its effectiveness without the need for ongoing intervention.
- Diabetes remission is more durable among individuals with normal BMI.
- Diabetes remission maintenance is primarily attributed to the reduction in the use of insulin or insulinotropic agent during the intervention period.

## Introduction

The notion of diabetes remission has been introduced in the 21st century and demonstrated through clinical research to be an attainable objective (1). The study of “Intermittent Calorie-restricted Diet on Type 2 Diabetes Remission” provided evidence that true low-calorie food interventions (as opposed to meal replacements) could lead to diabetes remission (2). In the present study, it was reported that patients with up to 11 years of type 2 diabetes mellitus (T2DM), including those on insulin therapy, achieved a diabetes remission rate of 47.2% after three months of CMNT intervention (six 15-day cycles, each consisting of five days of low-calorie intake [840 kcal/day] followed by 10 days of ad libitum eating). Moreover, obese T2DM participants with a disease duration of 6 years and no insulin use received 3–5 months of energy restriction followed by ongoing weight maintenance intervention, with 46% of the intervention group achieving remission in the first year (3). Remission has been regarded as the primary objective in the management and care of T2DM (4).

Following the demonstration of the feasibility of diabetes remission, the focus has now shifted to the achievement of long-term remission and the exploration of its influencing factors (5, 6). In the DIRECT follow-up study, some participants in the intervention group continued to participate in a 3-year Rescue Plan for weight regain after the initial weight reduction phase and the subsequent 2-year weight maintenance intervention. The findings demonstrated that the diabetes remission rate was sustained at 13% in the fifth year, while participants in the intervention group who did not adhere to the Rescue Plan exhibited a remission rate of only 3% (7). One reason why remission cannot be sustained is weight regain. Another possible reason is that, even after intervention, the participants’ BMI in the DIRECT study was still far above normal levels. In the CMNT study, participants in the intervention group did not receive any further treatment for one year after the three-month treatment period. In the one-year follow-up, 44.4% of participants from the intervention group were in remission for diabetes (2). This may be because the average baseline BMI of the intervention group in the CMNT study was below 24 kg/m², returning to normal levels after the intervention with no weight gain occurring within the one-year follow-up period. As a result, BMI level after remission may be a key factor in maintaining remission (8).

The CMNT diet has demonstrated the potential to achieve remission that does not require ongoing intervention to maintain. Nevertheless, a one-year period of observation is inadequate for determining the feasibility of maintaining remission, and a more protracted period of observation is required to confirm this. This study will observe the maintenance of remission in individuals with a lower BMI who are not receiving ongoing treatment through a three-year follow-up CMNT study.

## Methods

### Study design and data sources

The “Intermittent Calorie-restricted Diet on Type 2 Diabetes Remission” study was a randomized controlled trial with parallel-design and open-label. Participants were randomized in a 1:1 ratio to take part in either the CMNT or control group. Statistical analysis was conducted by using SAS/Base statistical software (SAS Inc.) and the random numbers were generated by the trial statistician. Ethical approval was obtained from the Chinese Ethics Committee of Registering Clinical Trials (ChiECRCT20200235), and the trail was conducted in local primary care centers. The detailed recruitment methods, study design, planned analyses, baseline characteristics, and primary study findings during the one-year follow-up have been published elsewhere (2).

Eligible participants were those diagnosed with T2DM according to the 1999 World Health Organization recommendations, aged 18 to 75 years old, with a body mass index (BMI) between 18 and 35 kg/m² who took oral hypoglycemic drugs such as sulfonylureas, meglitinides, metformin, dipeptidyl peptidase-4 inhibitors (DPP4i), glucagon like peptide-1 agonist (GLP1-RA), thiazolidinedione and/or insulin injections. Individuals were excluded if they had a diabetes type other than T2DM; had used other medications that may affect blood glucose levels within 2 months; had nonspecific dosages of antihypertensive or lipid-regulating drugs before screening; had New York Heart Association (NYHA) class III or IV cardiac insufficiency; had unstable angina; had a history of coronary artery bypass grafting, myocardial infarction, or stent implantation, two or more episodes of severe hypoglycemia in the past six years; had a history of eating disorders, systolic blood pressure ≥160 mmHg and/or diastolic blood pressure ≥100 mmHg; were pregnancy or breastfeeding, or any condition deemed unsuitable for participation by the investigator.

### Procedures

The intervention for the CMNT group consisted of six cycles of 15 intervention days. Each cycle included five modified fasting days (~840 kcal/day) using CMNT kits (ingredients see **Supplementary Table 1**), followed by 10 days of usual diet, as was done for the entire control group, which followed the Dietary Guidelines for Diabetes in China (2017 Edition). Following the three-month intervention period, both groups resumed their usual diet and continued receiving routine diabetes care in accordance with best practices.

Participants from both the CMNT and control groups who completed the intervention and the one-year follow-up were included in the three-year follow-up. During the follow-up, both groups adhered to the Dietary Guidelines for Diabetes in China (2017 Edition) and were allowed to consume foods of their choice. During the three-year follow-up, participants in the CMNT group who were not receiving ongoing intervention, and were not in remission or relapsed, received primary medical care as needed. All participants received optimal primary healthcare, with follow-ups every three month to document medication use, diet, physical activity, lifestyle habits, and self-monitored blood glucose and blood pressure. The medication regimen and dose-adjustment procedures for the participants remained identical to those described in our previously published study (2). General practitioners reviewed these data and provided individualized treatment recommendations (including medication adjustments). Participants underwent an annual physical examination at their local centers.

### Outcomes

The primary outcome was diabetes remission, defined as an HbA1c level of less than 48 mmol/mol (6.5%) for at least three months without the use of antidiabetic medication. Secondary outcomes included changes in HbA1c, fasting blood glucose (FBG), blood pressure, BMI, and quality of life. All anthropometric and biochemical measurements were collected during the three-year follow-up. Quality of life was evaluated using the EuroQol 5 Dimensions (EQ-5D-5L) scale. Clinical complications, including cardiovascular events, diabetic retinopathy, diabetic foot, hypertension, diabetic kidney disease, and all-cause mortality, were recorded throughout the follow-up period.

Diabetic retinopathy was diagnosed based on retinal examination findings through dilated fundus photography or optical coherence tomography (OCT). Diagnosis criteria included the presence of microaneurysms, retinal hemorrhages, exudates, or evidence of advanced stages such as neovascularization or macular edema (9). Diagnosis of diabetic foot was based on clinical assessment, including a history of neuropathy symptoms, peripheral arterial disease, and foot ulceration. Neuropathy was confirmed using a 10-g monofilament test and vibration perception threshold testing. Peripheral arterial disease was assessed via ankle-brachial index (ABI < 0.9) or Doppler ultrasound. Foot ulcers were classified using the Wagner or Texas classification systems (10). Hypertension was defined as systolic blood pressure (SBP) ≥ 140 mmHg and/or diastolic blood pressure (DBP) ≥ 90 mmHg on two separate occasions, or the use of antihypertensive medications (11). Diabetic kidney disease was diagnosed based on persistently elevated urinary albumin-to-creatinine ratio (UACR ≥ 30 mg/g) in at least two of three samples over a 3-month period and/or a sustained estimated glomerular filtration rate (eGFR) < 60 mL/min/1.73 m² (12).

### Statistical analysis

Clinical characteristics, including HbA1c, FBG, BMI, blood pressure, medication use, and quality of life scores, were recorded for all participants. All statistical analyses were performed by using the R software (version 4.3.1). Normality was first evaluated for all continuous variables using the Shapiro-Wilk test. Continuous variables with a normal distribution were summarized as mean ± SD and compared between groups using the independent-samples t-test. Variables that departed from normality were presented as median (inter-quartile range, IQR) and were compared with the Wilcoxon rank-sum test. Categorical data were reported as frequency (percentage) and analyzed with the chi-square test. Logistic regression and multinomial logistic regression were used to investigate the associations between clinical variables and remission and to identify key factors influencing remission. A p-value of less than 0.05 was considered statistically significant.

## Results

**Figure 1:**
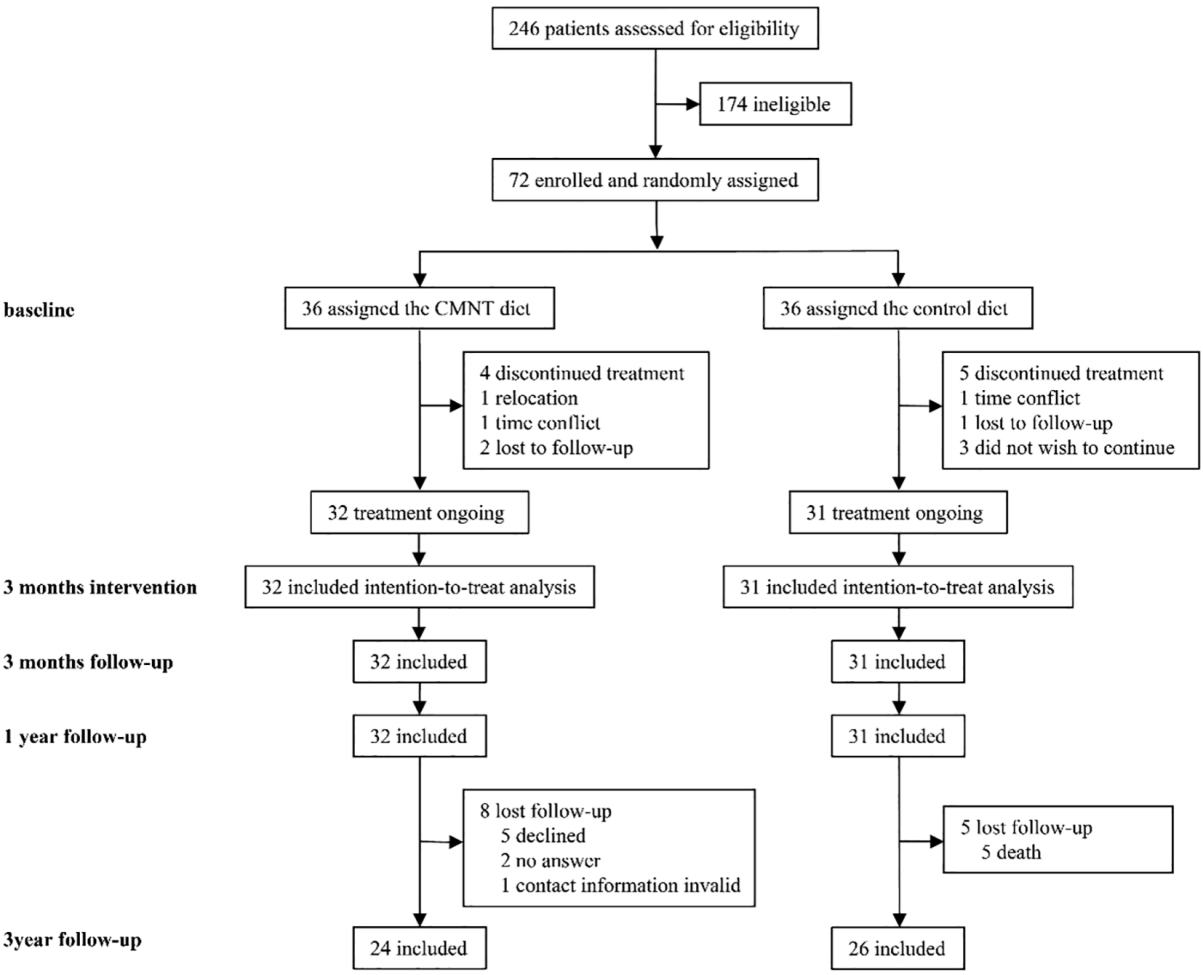
Trial profile.

From January 2, 2019 to June 2, 2020, 246 potential participants were recruited, among whom 72 were enrolled in the study (36 in each group). In the end, 32 participants in the CMNT group and 31 in the control group completed the intervention and the one-year follow-up (**Figure 1**). 24 of 32 participants (75%) in the CMNT group and 26 of 31 participants (84%) in the control group attended the 3-year- follow-up. In the CMNT group, 8 participants did not attend the three-year follow-up, including 5 who declined to participate, 2 who could not be contacted, and 1 whose contact information was unavailable. In the control group, 5 participants were lost to follow-up due to death (3 in 2022 and 2 in 2023) (**Figure 1**). Due to the high rate of missing data, all subsequent results are presented after multiple imputation to minimize potential bias. As a result, the remission rates and individual outcomes reported here may differ slightly from those in previous studies. It should also be emphasized that all reported outcomes, including remission rates, represent per-protocol estimates and do not reflect the overall treatment efficacy.

Baseline characteristics were compared between participants who completed and those who failed to complete the 3-year follow-up in the control group, except for a difference in quality-of-life scores, no significant inter-group differences were observed for any other parameter (**Supplementary Table 2**). Likewise, within the CMNT group, none of the baseline indices differed significantly between completers and non-completers. Furthermore, baseline parameters were compared between control and CMNT participants who completed the 3-year follow-up, while no significant between-group differences were identified (**Supplementary Table 2**).

During the 3-month follow-up, 17 participants in the CMNT group and 1 in the control group had achieved diabetes remission. In the first year, 16 CMNT participants remained in remission (they all had already remitted in the third months), whereas no control participant did. In the third year, 14 CMNT participants were still in remission (11 had already remitted in the first year and 3 were newly identified in the second year follow-up with drug withdrawal); the control group continued to show no remissions (**Table 1**). It is notable that none of the 11 who maintained remission in CMNT group used any antihyperglycaemic medication or received any CMNT diet intervention during the follow-up period. Significant differences in remission rates between the CMNT and control groups were observed in the 3^rd^ month, 1^st^ year, and 3^rd^ year (chi-square test).

**Table 1:** Diabetes remission at 3-month, 1-year, and 3-year follow-up.

Follow-up time	Control (n=31)			CMNT (n=32)		
	Remission	Maintain remission	New remission	Remission	Maintain remission	New remission
3 month follow-up	0	0	1	0	0	17
1 year follow-up	1	0	0	16	16	0
3 year follow-up	0	0	0	14	11	3

**Figure 2:**
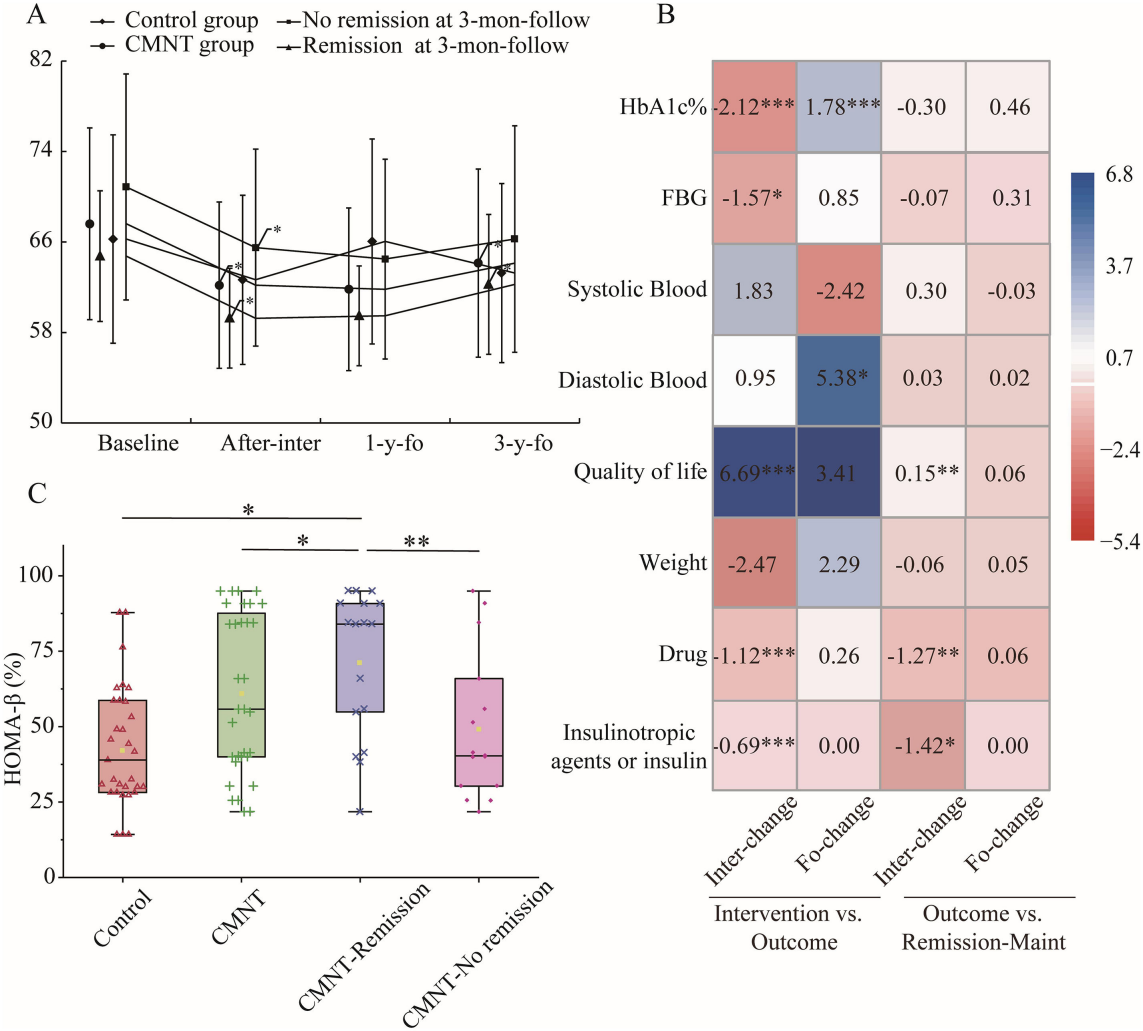
Analysis of factors associated with maintenance of diabetes remission. **(A)** Weight trends at baseline, post-intervention, 1-year, and 3-year follow-up. *p < 0.05, a significant difference between baseline and post-intervention, or a significant difference between 1-year and 3-years follow-up. Data points are not aligned with actual point in time for readability. **(B)** Effects of CMNT intervention on clinical outcome indicators (linear regression analyses) and the influence of these indicators on remission maintenance (logistic regression analyses). “Inter-change” denotes the change in each parameter during the intervention period; “Fo-change” denotes the change during the follow-up period. “Intervention vs Outcome” illustrates the association between CMNT intervention and the change in each parameter over both the intervention and follow-up phases. “Outcome vs Remission Maint” depicts the relationship between the change in each parameter during the intervention and follow-up phases and the maintenance of remission. Positive coefficients are depicted in shades of blue, while negative coefficients are shown in shades of red. The intensity of the color reflects the magnitude of the effect. Statistical significance is indicated directly on the heatmap: *p < 0.05, **p < 0.01, and ***p < 0.001. **(C)** HOMA-β level at the 3-years follow-up. Data are presented as box plots, showing the median, interquartile range (IQR), and overall distribution. *P < 0.05, **P < 0.01.

Although body weight increased in the CMNT group in the 3-year follow-up, the average BMI remained below 24 kg/m^2^ (**Table 2**). Subgroup analyses showed differences in weight trends between participants in the CMNT group who achieved remission during the 3-month follow-up and those who did not (**Figure 2A**). By the (end of) the 3-year follow-up, participants in the remission subgroup had regained weight, reaching levels comparable to baseline. In contrast, body weight remained essentially unchanged in the non-remission group by the end of the 3-year follow-up. However, it should be noted that the baseline body weight in the non-remission subgroup was significantly higher than that in the remission subgroup (P < 0.05) (**Supplementary Table 3**). These variations in body weight occurred despite no significant difference in calorie intake between the two groups (P = 0.093) (**Supplementary Table 4**).

**Table 2:** Clinical outcomes during 3-year follow-up.

Outcome	n	mran(SD)					Difference between 1 and 3 year follow-up	Difference between CMNT and control	
		Baseline	After-inter	1 year follow-up	3 year follow-up	Change between 1 and 3 year follow-up		3 year follow-up	Change between 1 and 3 year follow-up
							p^a^	p^b^	p^c^
Sex, male								0.359	
Control	31	20(65%)							
CMNT	32	25(78%)							
Age, year								0.929	
Control	31	50(41.0, 66.5)							
CMNT	32	52(47.8, 58.0)							
disease								0.933	
Control	31	8(4, 8)							
CMNT	32	7(4, 8)							
HbA1c, %								0.338	<0.001
Control	31	7.16 (6.29, 8.32)	7.32 (6.62, 9.03)	7.67 ± 1.04	6.76 ± 0.93	-0.92 ± 1.06	<0.001		
CMNT	32	7.07 (6.48, 7.87)	5.90 (5.48, 6.2)	6.1 (5.60, 6.43)	6.45 (6.18, 6.80)	0.27 ± 0.96	0.024		
FBG, mmol/L								0.068	0.117
Control	31	6.90 (6.285, 9.05)	6.90 (5.97, 9.04)	7.46 ± 1.75	7.35 ± 1.81	0.02 (-1.18,0.74)	0.762		
CMNT	32	7.00 (6.15, 9.725)	6.15 (5.4, 6.925)	6.10 (5.35, 6.54)	6.43 (5.71, 7.38)	0.44 (-0.35,1.36)	0.028		
Systolic Blood pressure, mmhg								0.106	0.298
Control	31	135 (125.00, 138.50)	130 (124.50, 136.00)	129.61 ± 9.12	132.74 ± 7.37	3.13 ± 12.65	0.178		
CMNT	32	129.41 ± 9.20	128.66 ± 9.25	128.81 ± 6.69	128.78 ± 11.38	-0.03 ± 11.19	0.987		
Diastolic Blood pressure, mmhg								<0.001	0.005
Control	31	83.06 ± 6.21	81.71 ± 6.87	81.55 ± 6.33	77.68 ± 6.97	-3.87 ± 8.47	0.016		
CMNT	32	84.41 ± 5.64	84.00 ± 4.54	83.59 ± 4.70	85.34 ± 6.44	1.75 ± 6.73	0.151		
BMI, kg/m^2^								0.567	0.002
Control	31	24.65 ± 3.18	23.27 ± 2.24	24.58 ± 3.12	23.49 ± 2.35	-1.09 ± 2.4	0.017		
CMNT	32	24.44 ± 3.21	22.48 ± 2.79	22.36 ± 2.84	23.13 ± 2.68	0.77 ± 2.04	0.041		
Weight, kg								0.669	0.001
Control	31	66.26 ± 9.20	62.66 ± 7.48	66.05 ± 9.06	63.25 ± 7.92	-2.8 ± 6.35	0.020		
CMNT	32	67.61 ± 8.48	62.18 ± 7.36	61.83 ± 7.19	64.13 ± 8.31	2.31 ± 5.61	0.027		
EQ-5D scale score								0.008	0.733
Control	31	75.00 (72.50, 85.00)	75.00 (70.00, 80.00)	74.84 ± 7.69	76.61 ± 10.44	1.77 ± 13.88	0.482		
CMNT	32	75.00 (75.00, 80.00)	80.00 (75.00, 85.00)	82.34 ± 6.84	85.16 ± 7.67	2.81 ± 9.75	0.113		

p^a^, significant difference between 1 and 3 year follow-up in CMNT group or control group. p^b^, significant difference between CMNT group and control group at 3-years follow-up.p^c^, significant difference for change of 1 to 3 year follow-up between CMNT group and control group.

Separate linear regression analyses were performed to further investigate the mediating factors influencing remission maintenance under CMNT intervention. The analysis assessed the effects of CMNT intervention on observational indicators during the intervention and follow-up periods, as well as the effects of these indicators on remission maintenance (**Figure 2B**). The maintenance of remission was found to be positively correlated with the extent of withdrawal of glucose-lowering medications (estimate = -1.42, 95% CI -2.68 to -0.30, P = 0.016) (specifically insulin or insulin- otropic agents, independent of other factors (estimate = -1.28, 95% CI -2.17 to -0.53, P = 0.002)) through the intervention, and was independent of weight changes during both the intervention (estimate = -0.06, 95% CI -0.22 to 0.05, P = 0.371) and follow-up (estimate = 0.05, 95% CI -0.06 to 0.16, P = 0.330) periods. CMNT intervention was negatively correlated with HbA1c (estimate = -2.12, 95% CI -2.93 to -1.30, P < 0.001), fasting glucose (estimate = -1.59, 95% CI -2.97 to -0.17, P = 0.029), BMI (estimate = -2.09, 95% CI -2.80 to -1.38, P < 0.001) and medication use (estimate = -1.20, 95% CI -1.47 to -0.77, P < 0.001) (insulin or insulin secretagogue (estimate = -0.69 CI -0.90 to -0.48, P < 0.001)) and positively correlated with quality of life (estimate = 6.69 CI 3.58 to 9.80, PP < 0.001). It should also be emphasized that the relationships among CMNT intervention, changes in the observed parameters, and the maintenance of diabetes remission are potentially confounded by collinearity and circular causation.

During the 3-year follow-up, fasting insulin data were available for a subset of participants, including 20 from the control group and 9 from the CMNT group who achieved remission in the 3-month follow-up and 7 from the CMNT group who did not achieve remission. Analysis revealed that participants in the remission group had significantly higher HOMA-β% levels compared to both the non-remission group and the control group (**Figure 2C**).

Comparisons between the imputed and complete-case analysis revealed three discrepant findings (**Supplementary Table 5**). Before imputation, HbA1c in the CMNT group did not differ significantly between the 3-year and 1-year visits; however, the change became statistically significant after imputation. Analogous patterns were observed for (1) the 3-year fasting plasma glucose difference between the remission and non-remission subgroups within the CMNT group, and (2) the baseline BMI difference between these two subgroups. Conversely, the 1-year follow up HbA1c comparison between remission and non-remission subgroups in the CMNT group was not significant after imputation, whereas it had been significant in the complete-case analysis. All other endpoints yielded identical inferential conclusions under the two analytical strategies.

## Discussion

This study demonstrates that diabetes remission achieved through a 3-month CMNT diet intervention can be maintained over 3 years without ongoing pharmacological or diet interventions. Notably, 64.7% (n = 17, of which 11 maintained remission) of participants in the CMNT group who had achieved remission by the 3rd-month post-intervention, maintained it for 3 years under ad libitum dietary conditions, highlighting the potential of this approach to alleviate the burden of long-term disease management. Different from previous studies, such as the DIRECT trial, which required ongoing support to mitigate weight regain and preserve remission, our findings underscore the feasibility of achieving long-term remission without structured maintenance strategies. Although this remission requires maintaining a normal BMI, the findings nonetheless challenge the conventional assumption that prolonged intervention is indispensable.

Long-term weight maintenance interventions have been used to support long-term diabetes remission due to the effects of weight regain, but their outcomes remain suboptimal (13). In the DIRECT follow-up study, for instance, even with low-intensity rescue programs provided over three to five years, many participants experienced weight regain, leading to a decline in remission rates from 51% to 13% (7). The CMNT diet intervention resulted in weight loss, while weight regain was also observed and primarily among participants who achieved remission. However, relapse was less frequent, even though only general dietary recommendations was provided. Linear regression analysis further revealed that maintenance of remission was not affected by weight changes during the follow-up. In energy-restricted diets, weight loss and weight regain are common phenomena, including participants from CMNT. However, given that the baseline BMI of the CMNT group was not elevated (approximately 25 kg/m²) and further decreased following the intervention, the average BMI remained below 24 kg/m² even after weight regain. In DIRECT study, a significant proportion of the participants exhibited a BMI greater than 30 kg/m², and even subsequent to the intervention, the BMI remained greater than 27 kg/m² (7). This may provide a reason for the high diabetes remission rate observed in the three-year follow-up after CMNT intervention in this study.

The Dual-Cycle Hypothesis of T2DM suggests that the interaction between hepatic insulin resistance caused by liver fat accumulation and β-cell dysfunction induced by pancreatic exposure to hypertriglyceridemia drives the onset and progression of T2DM (14). Among participants in the CMNT group who maintained remission, the BMI was 23.9 kg/m² at baseline and 21.7 kg/m² after intervention. In the three newly added subjects whose diabetes was in remission, the intervention was followed by the use of only one hypoglycaemic agent and concomitant HbA1c of less than 6%. Although these participants’ weight had increased during the follow-up, all but one participant had a BMI below 25 kg/m²in the 3 -year follow-up. A BMI below 25 kg/m² is generally associated with a lower risk of developing T2DM and is often indicative of relatively limited hepatic fat accumulation (15). This phenotype may partly explain the favorable conditions that support diabetes remission maintenance in our cohort. However, this assumption requires further validation with direct measurements of hepatic steatosis, as the current study lacks corresponding liver fat data. Furthermore, the dietary recommendations stipulated within the Dietary Guidelines for Diabetes in China, which were to be adhered to by subjects during the follow-up period, including a low GL diet, management of staple foods, and increased vegetable intake, also demonstrated glycaemic control benefits. Overall, maintaining diabetes remission may be partly attributable to sustaining a normal BMI together with adherence to healthy dietary patterns.

As long as the pancreas can compensate by increasing insulin secretion, plasma glucose control can be maintained in both muscle and liver, even in the presence of insulin resistance (14). Some studies have demonstrated that in the absence of weight loss, such as in the context of intensive insulin therapy (16), GLP-1 therapy (17), or other interventions, diabetes remission can be achieved. In this follow-up study, we evaluated the impact of the CMNT diet intervention on participants’ use of insulinotropic agents and insulin. Results showed a 86.7% reduction in usage (**Supplementary Table 6**) among participants in the CMNT group during the intervention period, with no subsequent increase observed throughout the follow-up period. These findings may reflect an improvement in the participants’ endogenous insulin secretory capacity, potentially reducing their reliance on insulin-secretagogue medications or exogenous insulin therapy. Furthermore, animal studies have demonstrated that CMNT can promote β-cell proliferation and enhance insulin secretion in mice, supporting a possible mechanistic basis for the observed clinical benefits (18). In addition, we observed that participants in the CMNT group who remained in remission in the 3-year follow-up exhibited higher HOMA-β levels compared with those who did not (**Figure 2C**). Although this observation is based on a relatively small sample size and requires confirmation in larger and more rigorous studies, it remains a noteworthy finding. Taken together, these observations may indicate a potential role of CMNT in supporting metabolic improvements that favor diabetes remission, possibly through modest enhancements in endogenous insulin secretion and β-cell function.

The majority of individuals with diabetes express a strong desire to achieve autonomy to live their lives as they choose, free from the need for medication, insulin, or restrictive diets, and without the requirement for strenuous exercise (19, 20). This is a goal that their physicians had previously indicated unattainable, even if medication was ceased, particularly in individuals dependent on insulin (11, 21). Nevertheless, the necessity of long-term disease management imposes varying degrees of psychological stress on patients (22). In this study, those who achieved remission in CMNT group were not only free from the need for antidiabetic medications, but also did not require interventions that differed from their normal routine. These benefits were reflected in the markedly superior quality of life in the CMNT group in comparison to the control group. Diabetes-related complications are common and can impact multiple organ systems, leading to increased mortality, blindness and renal failure (23). Despite the absence of ongoing intervention during the 3-year follow-up period, the CMNT group exhibited a five-fold reduction in hospitalisation rates and a three-fold reduction in complication rates compared to the control group (**Supplementary Table 7**). In summary, CMNT-induced remission could enable some individuals to reduce their reliance on pharmacological therapy and may be associated with improvements in disease-related burden, although further studies are needed to confirm long-term effects without ongoing intervention.

It is important to acknowledge the limitations of this study. On the one hand, the sample size was relatively small, and the three-year follow-up period was not sufficiently long. Future research with larger cohorts and longer follow-up duration is needed to confirm these results and further explore the long-term impact of CMNT interventions on diabetes remission. On the other hand, CMNT needs to be validated for its effectiveness in remission diabetes in people with higher BMI, and its long-term benefits need to be explored. It should also be noted that remission status in the present study was defined solely on the basis of annual HbA1c measurements and thus reflects the remission status only at the time of assessment. Owing to the absence of additional day-to-day normoglycaemic data, the findings do not provide evidence for sustained metabolic stability.

In conclusion, this study highlights the CMNT diet as a promising intervention for achieving long-term diabetes remission. It is noteworthy that this remission was maintained without the requirement for ongoing pharmacological or dietary intervention, provided that a normal BMI was preserved. Meanwhile, the preservation of pancreatic β-cell function is also an important contributing factor. This diet-focused approach demonstrates the potential to reduce reliance on insulin and insulinotropic agents while improving quality of life and minimizing the burden of long-term management.

## Data Availability

The original contributions presented in the study are included in the article/**Supplementary Material**. Further inquiries can be directed to the corresponding author/s.
