# Supplementary material for: Maintained diabetes remission among normal BMI individuals achieved without ongoing intervention: a three-year follow-up study of intermittent calorie restriction
Source: Front Endocrinol (Lausanne). 2026 Jan 28;16:1733840. doi: 10.3389/fendo.2025.1733840 (PMC12890618; doi:10.3389/fendo.2025.1733840)
Supplement: Supplementary file 2 [file Table1.docx]

**Supplementary Table 1. Chinese Medical Nutrition Therapy diet calorie content and ingredients**

| **Calorie information** | | | | | | | |
| --- | --- | --- | --- | --- | --- | --- | --- |
|  | | **Solid beverages** | | **Fruit and vegetable gruel** | **Composite nutritional rice** | **Meal replacement biscuit** |  |
| Energy density (kcal/100g) | | 576.24 | | 533.22 | 358.75 | 489.96 |  |
| Protein (g/100g) | | 7.20 | | 3.40 | 10.50 | 7.10 |  |
| Protein % | | 5.28 | | 2.52 | 11.86 | 5.97 |  |
| Fat (g/100g) | | 50.00 | | 30.80 | 1.80 | 18.20 |  |
| Fat % | | 84.09 | | 52.39 | 4.66 | 35.13 |  |
| Carbohydrates (g/100g) | | 14.50 | | 60.80 | 73.90 | 70.00 |  |
| Carbohydrates % | | 10.63 | | 45.09 | 83.48 | 58.90 |  |
| Fiber (g/100g) | | 23.90 | | - | - | 8.20 |  |
| Sodium (mg/100g) | | 63.00 | | 95.00 | 41.20 | 264.00 |  |
| **Diet ingredients** | | | | | | | |
|  | **Daily intake** | | **Note** | | | |  |
| **Breakfast** | | | | | | |  |
| Fruit and vegetable gruel | 50 g  (266.61 kcal) | | Fresh pumpkins, pumpkin seed kernel oil, maltodextrins, isomalto-oligosaccharide, casein, resistant dextrin, sodium ascorbate, potassium citrate, monoglycerides and diglycerides of fatty acids esters, vitamin E, tea polyphenols, and silicon dioxide | | | |  |
| **Lunch** | | | | | | |  |
| Solid beverages | 25 g  (144.06 kcal) | | Pumpkin seed kernel oil, isomalto-oligosaccharide, casein, resistant dextrin, sodium ascorbate, potassium citrate, Monoglycerides and diglycerides of fatty acids esters, vitamin E, tea polyphenols, and silicon dioxide | | | |  |
| Composite nutritional rice | 60 g  (215.25 kcal) | | Rice, millet, corn, buckwheat, quinoa, oat, spinach powders, lily root flour, cucumber powders, mushroom powder, wheat dietary fiber, bitter melon, pumpkins, potato, purple potato, sweet potato, mung bean, Konjac flour, inulin, *Fructus lyci, Ganoderma lucidum, Folium Mori, Poria cocos, Dioscorea opposita Thunb*. (Chinese yam), *Radix puerariae, Cordyceps militaris, Momordica grosvenori*, and edible refined salt | | | |  |
| **Dinner** | | | | | | |  |
| Solid beverages | 25 g  (144.06 kcal) | | Fresh pumpkin seed kernel oil, isomalto-oligosaccharide, casein, resistant dextrin, sodium ascorbate, potassium citrate, mono-and diglycerides of fatty acids esters, vitamin E, tea polyphenols, and silicon dioxide | | | |  |
| Biscuit | 30 g  (146.99 kcal) | | Wheat flour, MAIKERENJIA, mix powder (quinoa, white kidney beans, wheat germ, azuki bean, black beans, yellow beans, *Liriopes radix*, glutinous rice, black rice, maize, round bract Plantago ovata husk powder, oat, buckwheat, Chinese yam, hawthorn, roselle, millet, brown rice, Chinese jujube, Chinese wolfberry, pecan nuts, chia seed, black sesame, white sesame, shiitake mushroom, Laminaria hyperborean, coffee), edible vegetable oils, potato protein, wheat dietary fiber powder, resistant dextrin, maltodextrin, and L-arabinose | | | |  |

**Supplementary Table 2 Baseline data of subjects who completed versus those who did not complete the 3-year follow-up in both the control and CMNT groups.**

|  | **Control n = 31** | | | **CMNT n = 32** | | | **Completed**  **Control vs CMNT** |
| --- | --- | --- | --- | --- | --- | --- | --- |
|  | **Completed n = 26** | **Incomplete n = 5** | **p** | **Completed n = 24** | **Incomplete n = 8** | **p** | **p** |
| Sex, n (%) |  |  | 0.133 |  |  | 0.646 | 0.321 |
| Male | 15 (58%) | 5 (100%) |  | 18 (75) | 7 (88) |  |  |
| Female | 11 (42%) | 0 (0%) |  | 6 (25) | 1 (12) |  |  |
| Age, year | 58.0 (42.5, 66.75) | 41.0 (40.0, 41.0) | 0.052 | 51.50 ± 7.11 | 56.62 ± 7.25 | 0.108 | 0.272 |
| Disease, year | 8 (4, 8) | 6 (5, 8) | 0.870 | 8（5.5, 8） | 7 (4., 7.3) | 0.452 | 0.728 |
| High, m | 1.64 ± 0.08 | 1.66 ± 0.08 | 0.531 | 1.66 ± 0.07 | 1.68 ± 0.04 | 0.331 | 0.250 |
| HbA1c, % | 7.63 ± 1.39 | 6.84 ± 1.24 | 0.252 | 7.25 (6.47, 8.85) | 6.61 (6.42, 7.05) | 0.170 | 0.992 |
| FBG, mmol/L | 7.54 (6.38, 9.12) | 6.35 (5.70, 6.51) | 0.126 | 7.4 (5.97, 10.42) | 6.92 (6.50, 7.62) | 0.617 | 0.961 |
| Systolic Blood pressure, mmhg | 132.42 ± 11.34 | 134.6 ± 6.69 | 0.573 | 130 (123, 134.25) | 124.5 (120, 127) | 0.106 | 0.607 |
| Diastolic Blood pressure, mmhg | 82.35 ± 6.13 | 86.8 ± 5.76 | 0.169 | 82.5 (80.0, 88.5) | 81.5 (80.0, 85.5) | 0.625 | 0.470 |
| Weight, kg | 66.45 ± 8.71 | 65.28 ± 12.64 | 0.851 | 68.42 ± 8.64 | 65.17 ± 7.99 | 0.347 | 0.425 |
| BMI, kg/m2 | 24.88 ± 3.19 | 23.47 ± 3.15 | 0.398 | 24.88 ± 3.28 | 23.12 ± 2.76 | 0.159 | 0.998 |
| Glucose-lowering medications, n (%) |  |  | 0.835 |  |  | 1.000 | 0.873 |
| 1 | 9 (35) | 1 (20) |  | 10 (42) | 3 (38) |  |  |
| 2 | 10 (38) | 3 (60) |  | 8 (33) | 3 (38) |  |  |
| 3 | 7 (27) | 1 (20) |  | 6 (25) | 2 (25) |  |  |
| EQ-5D scale score | 78.85±6.68 | 72.00±2.74 | 0.030 | 76.67±7.02 | 74.75±8.31 | 0.719 | 0.328 |

|  |
| --- |

**Supplementary Table 3 Clinical outcomes during 3-year follow-up. Superscripts indicate significant differences as follows: (^*^), CMNT group with remission vs. non-remission at 3-month follow-up (*p* < 0.05).**

|  |  | **CMNT group** | |
| --- | --- | --- | --- |
|  |  | **Yes (n=17)** | **No (n=15)** |
|  |  | **mean±SD/median(Q1,Q3)** | **mean±SD/median(Q1,Q3)** |
| gender, n (%) |  |  |  |
|  | male | 11 (73%) | 14 (82%) |
|  | female | 4 (27%) | 3 (18%) |
| age,y |  | 54.20 ± 8.31 | 51.53 ± 6.44 |
| Duration of type 2 diabetes, y |  | 7 (3.5, 8) | 8 (6, 8) |
| HbA1c,% | baseline | 6.80 (6.20, 7.90) | 7.2 (6.58, 7.73) |
|  | After-inter | 5.86 (5.40, 6.50) | 5.9 (5.75, 6.07) |
|  | 1-y-fo | 6.00 (5.60, 6.30) | 6.61 ± 1.37 |
|  | 3-y-fo | 6.21 (5.90, 6.60) | 6.60 (6.45, 6.8)^*^ |
| FBG,mmol/L | baseline | 6.60 (5.90, 7.00) | 9.11 ± 2.90^*^ |
|  | After-inter | 6.00 (5.40, 6.60) | 6.59 ± 1.14 |
|  | 1-y-fo | 6.10 (5.20, 6.50) | 6.26 ± 1.10 |
|  | 3-y-fo | 6.10 (5.70, 6.88) | 7.28 ± 1.47^*^ |
| Systolic Blood pressure,mmhg | baseline | 126.53 ± 7.13 | 132.67 ± 10.39 |
|  | After-inter | 124.88 ± 9.45 | 132.93 ± 7.12^*^ |
|  | 1-y-fo | 129.00 (122.00, 131.00) | 131.53 ± 5.82^*^ |
|  | 3-y-fo | 127.29 ± 11.32 | 130.47 ± 11.59 |
| Diastolic Blood pressure,mmhg | baseline | 84.06 ± 5.72 | 82.00 (80.50, 88.50) |
|  | After-inter | 83.71 ± 3.55 | 83.00 (80.5, 88) |
|  | 1-y-fo | 82.94 ± 3.67 | 84.33 ± 5.70 |
|  | 3-y-fo | 83.53 ± 5.87 | 87.40 ± 6.62 |
| Weight, kg | baseline | 64.74 ± 5.77 | 70.87 ± 9.98^*^ |
|  | After-inter | 59.26 ± 4.40 | 65.49 ± 8.70^*^ |
|  | 1-y-fo | 59.47 ± 4.41 | 65.1 (61.2, 69.5) |
|  | 3-y-fo | 63.5 (60, 66.5) | 66.27 ± 10.00 |
| BMI,kg/m^2^ | baseline | 23.28 ± 2.64 | 25.75 ± 3.37^*^ |
|  | After-inter | 21.32 ± 2.23 | 23.78 ± 2.84^*^ |
|  | 1-y-fo | 21.39 ± 2.19 | 23.49 (21.50, 24.69)^*^ |
|  | 3-y-fo | 22.49 (21.26, 23.44) | 24.04 ± 3.06 |
| Quality of life | baseline | 75.00 (70.00, 80.00) | 80.00 (75.00, 80.00) |
|  | After-inter | 80.00 (75.00, 85.00) | 80.00 (77.50, 85.00) |
|  | 1-y-fo | 80.88 ± 5.07 | 84.00 ± 8.28 |
|  | 3-y-fo | 86.76 ± 7.28 | 83.33 ± 7.94 |

**Supplementary Table 4 Kilocalorie intake at 3 years follow-up. The China Food Composition Database that incorporates the Chinese Food Composition Table was employed to estimate the energy content of a daily meal. Data are daily means (SD）**

|  | **Groups** | **Dietary regimen** | **Kilocalorie** | ***p values*** |
| --- | --- | --- | --- | --- |
| 3 years follow-up | Control group (n=26) | Dietary Guidelines for Diabetes in China (2017 Edition) | 2167（221） | 0.093 |
|  | CMNT group (n=24) |  | 2259（336） |  |

**Supplementary Table 5 Clinical outcomes during 3-year follow-up (Complete-Case Analysis). Superscripts indicate significant differences as follows: (^a^) CMNT group vs. control group, (^b^) CMNT group with remission vs. non-remission at 3-month follow-up, (^c^) CMNT group without remission at 3-month follow-up vs. control group, (^d^) CMNT group with remission at 3-month follow-up vs. control group, and (^e^) 1 year follow up vs. 3 year follow up (*p* < 0.05). Double (^aa^) and triple (^aaa^) superscripts denote *p* < 0.01 and *p* < 0.001, respectively. The same notation applies for (^b^), (^c^), (^d^), and (^e^).**

|  |  |  | Control group | CMNT group | | CMNT group remission at 3-mon-follow | | | |
| --- | --- | --- | --- | --- | --- | --- | --- | --- | --- |
|  |  |  |  |  |  |  | Yes |  | No |
|  |  | n | mean±SD/median(Q1,Q3) | n | mean±SD/median(Q1,Q3) | n | mean±SD/median(Q1,Q3) | n | mean±SD/median(Q1,Q3) |
| gender, n (%) |  | 31 |  | 32 |  | 17 |  | 15 |  |
|  | male |  | 20 (65%) |  | 25 (78%) |  | 11 (73%) |  | 14 (82%) |
|  | female |  | 11 (35%) |  | 7 (22%) |  | 4 (27%) |  | 3 (18%) |
| age,y |  | 31 | 50 (41,66.5) | 32 | 52 (47.75,58) | 17 | 54.2 ± 8.31 | 15 | 51.53 ± 6.44 |
| Duration of type 2 diabetes, y |  | 31 | 8 (4,8) | 32 | 7 (4,8) | 17 | 7 (3.5, 8) | 15 | 8 (6, 8) |
| HbA1c,% | baseline | 31 | 7.16 (6.28,8.32) | 32 | 7.06 (6.47,7.87) | 17 | 7.2 (6.58,7.73) | 15 | 6.8 (6.2,7.9) |
|  | After-inter | 31 | 7.32 (6.62,9.02)aaa | 31 | 5.9 (5.47,6.2) | 17 | 5.82 ± 0.4ccc | 15 | 5.85 ± 0.89ddd |
|  | 1-y-fo | 30 | 7.8 (6.82,8.07)aaa | 30 | 6.1 (5.62,6.4) | 17 | 6.45 (6.12,7.09)bc | 15 | 5.9 (5.6,6.15)ddd |
|  | 3-y-fo | 26 | 6.57 (6.23,6.79)^eee^ | 24 | 6.3 (6.18,6.6) | 12 | 6.57 (6.38,6.97)b | 12 | 6.2 (6.05,6.3)dde |
| FBG,mmol/L | baseline | 31 | 6.9 (6.28,9.05) | 32 | 7 (6.15,9.72) | 17 | 9.4 (7,10.43)b | 15 | 6.6 (5.9,7) |
|  | After-inter | 31 | 6.9 (5.97,9.04)aa | 32 | 6.14 (5.4,6.93) | 17 | 6.59 ± 1.14 | 15 | 5.99 ± 1.01dd |
|  | 1-y-fo | 31 | 6.9 (6.2,8.4)aaa | 32 | 6.1 (5.35,6.54) | 17 | 6.26 ± 1.1c | 15 | 5.98 ± 0.88dd |
|  | 3-y-fo | 26 | 6.78 (6.5,7.26) | 24 | 6.6 (6.02,7.37)^e^ | 12 | 7 (6.39,8.16) | 12 | 6.34 (5.71,6.92) |
| Systolic Blood pressure,mmhg | baseline | 31 | 132.77 ± 10.67 | 32 | 129.41 ± 9.2 | 17 | 131 (126,134.5) | 15 | 125 (121,131)d |
|  | After-inter | 31 | 130.19 ± 9.57 | 32 | 128.66 ± 9.25 | 17 | 132.93 ± 7.12b | 15 | 124.88 ± 9.45 |
|  | 1-y-fo | 31 | 129.61 ± 9.12 | 30 | 129.1 ± 6.82 | 17 | 132.07 ± 5.64^b^ | 15 | 126.5 ± 6.85 |
|  | 3-y-fo | 26 | 132.73 ± 6.89 | 24 | 128.67 ± 10.89 | 12 | 131.83 ± 10.92 | 12 | 125.5 ± 10.34d |
| Diastolic Blood pressure,mmhg | baseline | 31 | 84 (80,88) | 32 | 81.5 (80,87) | 17 | 82 (80.5,88.5) | 15 | 81 (80,85) |
|  | After-inter | 31 | 83 (77,87.5) | 32 | 83.5 (80.75,87.25) | 17 | 83 (80.5,88) | 15 | 84 (81,85) |
|  | 1-y-fo | 31 | 82 (77,87) | 30 | 83 (81,85) | 17 | 83 (81,86.75) | 15 | 83 (80.75,84.25) |
|  | 3-y-fo | 26 | 78 (75.25,78.75)aaa^ee^ | 24 | 86 (81.75,90) | 12 | 86.92 ± 6.63cc | 12 | 83.92 ± 5.37d |
| Weight, kg | baseline | 31 | 66.25 ± 9.2 | 32 | 67.6 ± 8.47 | 17 | 70.85 ± 9.98b | 15 | 64.74 ± 5.77 |
|  | After-inter | 31 | 65.98 ± 9.31 | 32 | 62.18 ± 7.36 | 17 | 65.48 ± 8.7b | 15 | 59.26 ± 4.41dd |
|  | 1-y-fo | 31 | 66.05 ± 9.05a | 32 | 61.82 ± 7.19 | 17 | 64.48 ± 8.82 | 15 | 59.47 ± 4.41dd |
|  | 3-y-fo | 26 | 63.79 ± 10.34^e^ | 24 | 66.03 ± 9.19^e^ | 12 | 67.25 ± 11.59 | 12 | 64.25 (60,67.96)e |
| BMI,kg/m^2^ | baseline | 31 | 23.7 ± 2.65 | 32 | 24.7 ± 3.07 | 17 | 25.75 ± 3.37 | 15 | 23.78 ± 2.54 |
|  | After-inter | 31 | 23.57 ± 1.97 | 32 | 22.47 ± 2.79 | 17 | 23.78 ± 2.84b | 15 | 21.32 ± 2.23dd |
|  | 1-y-fo | 31 | 24.58 ± 3.12^aa^ | 32 | 22.36 ± 2.83 | 17 | 23.45 ± 3.14b | 15 | 21.39 ± 2.19ddd |
|  | 3-y-fo | 26 | 23.65 ± 2.14^eee^ | 24 | 23.93 ± 2.84^e^ | 12 | 24.45 ± 3.42 | 12 | 23.41 ± 2.14 |
| Quality of life | baseline | 30 | 75.00 (71.25, 83.75) | 32 | 75 (75, 80) | 17 | 80 (75, 80) | 15 | 75 (70, 80) |
|  | After-inter | 30 | 75 (70, 80)^aa^ | 32 | 80 (75, 85) | 17 | 80 (77.5, 85)^c^ | 15 | 80 (75, 85)^d^ |
|  | 1-y-fo | 30 | 70 (70, 80)^aaa^ | 32 | 80 (80, 85) | 17 | 80 (80, 92.5)^c^ | 15 | 80 (80, 85)^d^ |
|  | 3-y-fo | 25 | 75 (70, 85)^aa^ | 24 | 85 (80, 90) | 12 | 85 (80, 85)^c^ | 12 | 85 (83.75, 90)^d^e |

**Supplementary Table 6 Number of glucose-lowering medications, insulinotropic agents or insulin used in the CMNT group and control group during 3-year follow-up.**

|  |  | **Glucose-lowering medications，n (%)** | | | **Insulinotropic agents or insulin，n (%)** | | |
| --- | --- | --- | --- | --- | --- | --- | --- |
|  | **Number** | **Control (n=31)** | **CMNT (n=32)** | **p** | **Control (n=31)** | **CMNT (n=32)** | **p** |
| baseline |  |  |  | 0.763 |  |  | 0.015 |
|  | 1 | 10 (32%) | 13 (41%) |  | 14 (45%） | 5 (16%） |  |
|  | 2 | 13 (42%) | 11 (34%) |  | 14 (45%） | 25 (78%） |  |
|  | 3 | 8 (26%) | 8 (25%) |  | 3 (10%） | 2 (6%） |  |
| after intervention |  |  |  | <0.001 |  |  | 0.010 |
|  | 0 | 1 (3%) | 17 (53%) |  | 14 (45%） | 25 (78%） |  |
|  | 1 | 11 (35%) | 12 (38%) |  | 14 (45%） | 7 (22%） |  |
|  | 2 | 12 (39%) | 2 (6%) |  | 3 (10%） | 0 (0%） |  |
|  | 3 | 7 (23%) | 1 (3%) |  |  |  |  |
| 1 year follow-up |  |  |  | <0.001 |  |  | 0.010 |
|  | 0 | 1 (3%) | 16 (50%) |  | 14 (45%） | 25 (78%） |  |
|  | 1 | 11 (35%) | 13 (41%) |  | 14 (45%） | 7 (22%） |  |
|  | 2 | 12 (39%) | 2 (6%) |  | 3 (10%） | 0 (0%） |  |
|  | 3 | 7 (23%) | 1 (3%) |  |  |  |  |
| 3 year follow-up |  |  |  | <0.001 |  |  | 0.010 |
|  | 0 | 1 (3%) | 18 (56%) |  | 14 (45%） | 25 (78%） |  |
|  | 1 | 11 (35%) | 8 (25%) |  | 14 (45%） | 7 (22%） |  |
|  | 2 | 17 (55%) | 4 (12%) |  | 3 (10%） | 0 (0%） |  |
|  | 3 | 2 (6%) | 2 (6%) |  |  |  |  |

**Supplementary Table 7 All-cause mortality and major diabetes-related events during the three-year follow-up.**

|  | **control(n=31)** | **CMNT(n=32)** |
| --- | --- | --- |
| All-cause mortality |  |  |
|  | 5 (16.1%) | 0 (0.0%) |
| Total diabetic complications | 6 (18.75%) | 2 (6.25%) |
| Hypertension | 2 (6.5%) | 2 (6.25%) |
| Diabetic retinopathy | 1 (3.2%) | 0 (0.0%) |
| Diabetic foot | 1 (3.2%) | 0 (0.0%) |
| Cerebral infarction | 2 (6.5%) | 0 (0.0%) |
| Number of hospitalizations due to diabetes |  |  |
|  | 18(58.1%) | 4(12.5%) |
